# Publisher Correction: Dynamic structural states of ClpB involved in its disaggregation function

**DOI:** 10.1038/s41467-019-11204-x

**Published:** 2019-07-12

**Authors:** Takayuki Uchihashi, Yo-hei Watanabe, Yosuke Nakazaki, Takashi Yamasaki, Hiroki Watanabe, Takahiro Maruno, Kentaro Ishii, Susumu Uchiyama, Chihong Song, Kazuyoshi Murata, Ryota Iino, Toshio Ando

**Affiliations:** 1 Department of Physics and Structural Biology Research Center, Nagoya University, Chikusa-ku, Nagoya, 464-8602 Japan; 2 Department of Biology, Faculty of Science and Engineering, Konan University, Okamoto 8-9-1, Kobe, 658-8501 Japan; 3 Institute for Integrative Neurobiology, Konan University, Okamoto 8-9-1, Kobe, 658-8501 Japan; 4 Department of Physics, College of Science and Engineering, Kanazawa University, Kanazawa, 920-1192 Japan; 5 Department of Biotechnology, Graduate School of Engineering, Osaka University, Osaka, 565-0871 Japan; 6 Exploratory Research Center on Life and Living Systems (ExCELLS), National Institutes of Natural Sciences, Okazaki, Aichi 444-8787 Japan; 7 National Institute for Physiological Sciences, National Institutes of Natural Sciences, Okazaki, Aichi 444-8787 Japan; 8 Institute for Molecular Science, National Institutes of Natural Sciences, Okazaki, Aichi 444-8787 Japan; 9 Department of Functional Molecular Science, School of Physical Sciences, The Graduate University for Advanced Studies (SOKENDAI), Hayama, Kanagawa 240-0193 Japan; 10 Nano Life Science Institute (WPI-NanoLSI), Kanazawa University, Kanazawa, 920-1192 Japan

**Keywords:** Enzyme mechanisms, Atomic force microscopy, Atomic force microscopy, Single-molecule biophysics, Atomic force microscopy

Correction to: *Nature Communications* 10.1038/s41467-018-04587-w, published online 1 June 2018.

The original version of this Article did not acknowledge Ryota Iino as a corresponding author. This has now been corrected in both the PDF and HTML versions of the Article.

