# Supplementary material for: Dynamic structural states of ClpB involved in its disaggregation function
Source: Nat Commun. 2018 Jun 1;9:2147. doi: 10.1038/s41467-018-04587-w (PMC5984625; doi:10.1038/s41467-018-04587-w)
Supplement: Supplementary file 1 — Supplementary Information [file 41467_2018_4587_MOESM1_ESM.pdf]

Supplementary Information for

Dynamic Structural States of ClpB Involved in Its Disaggregation Function

Takayuki Uchihashi, Yo-hei Watanabe*, Yosuke Nakazaki, Takashi Yamasaki, Hiroki Watanabe, Takahiro Maruno, Kentaro Ishii, Susumu Uchiyama, Chihong Song, Kazuyoshi Murata, Ryota Iino*, Toshio Ando*

*Correspondence and requests for materials should be addressed to Y. W. (ywatanab@center.konan-u.ac.jp), R. I. (iino@ims.ac.jp), or T. A. (tando@staff.kanazawa-u.ac.jp)

This PDF includes:
Supplementary Figures 1-16
Supplementary Table 1-2

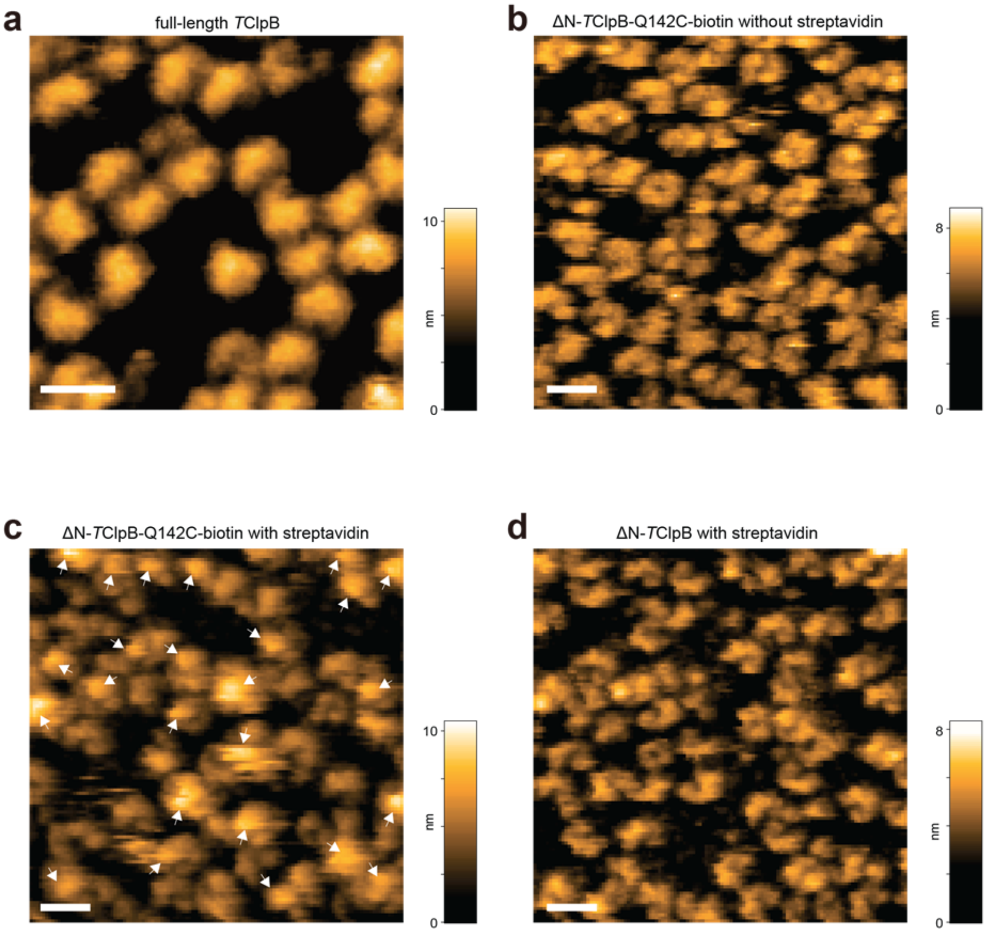

Supplementary Figure 1 | HS-AFM observation of full-length *T*ClpB and streptavidin binding to biotinylated ΔN-*T*ClpB. (**a**) HS-AFM image of full-length *T*ClpB acquired at 1 mM ATP after preincubation with 2 mM ATP at 55°C for 1 min. Scale bar, 20 nm. Z color bar, 0 to 10.7 nm. (**b**) HS-AFM image of biotinylated ΔN-*T*ClpB. Q142 residue positioned at the N-terminal face of ΔN-*T*ClpB was mutated into cysteine and reacted with biotin-PEAC5-maleimide. Scale bar, 20 nm. Z color bar, 0 to 8.9 nm. (**c**) HS-AFM image of biotinylated ΔN-*T*ClpB acquired ~10 min after the addition of streptavidin (0.4 μM) to the observation buffer. The white arrows indicate positions where streptavidin molecules seem to be bound. Scale bar, 20 nm. Z color bar, 0 to 10.6 nm. (**d**) HS-AFM image of ΔN-*T*ClpB acquired ~10 min after the addition of streptavidin (0.4 μM) to the observation buffer. This imaging was carried out as a negative-control experiment. Scale bar, 20 nm. Z color bar, 0 to 8.4 nm. No binding of streptavidin was observed.

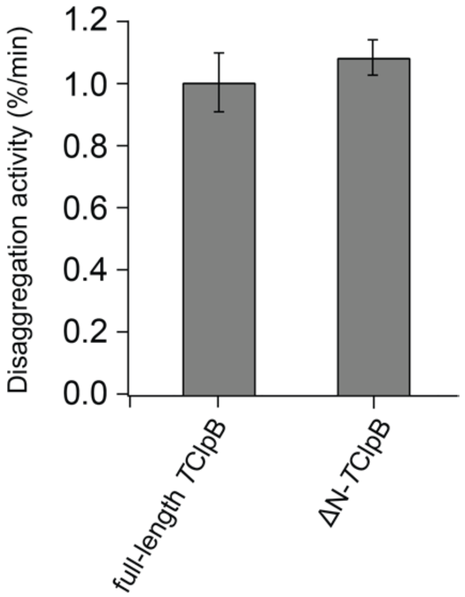

Supplementary Figure 2 | Disaggregation activity of full-length and ΔN-*T*ClpB. Each bar shows mean ± s.d. (n = 3). No difference in the disaggregation activity was observed between full-length and ΔN-*T*ClpB.

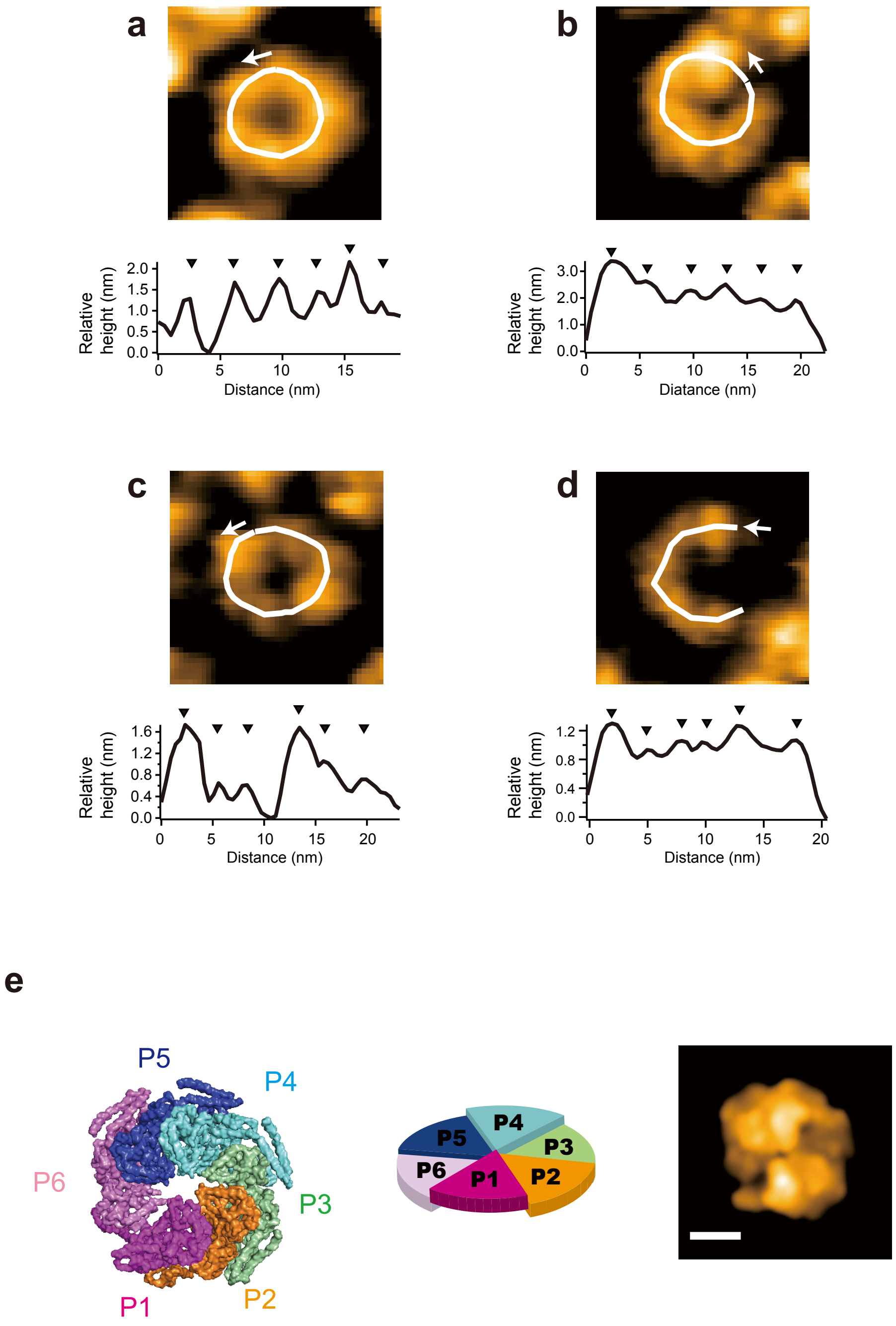

Supplementary Figure 3 | Cross-sectional analysis of typical oligomer forms. (**a–d, top**) Enlarged AFM images of four typical oligomer forms in the presence of 0.5 mM ATP: (**a**) round closed ring, (**b**) spiral ring, (**c**) twisted-half-spiral ring, and (**d**) open-ring form. We collectively refer to the spiral and twisted-half-spiral rings as the distorted closed ring. (**a–d, bottom**) Cross-sectional corrugation profiles (relative height) of the top surface of oligomers measured along the white lines overlaid on the images. The direction in which the cross-sectional profiles are taken is shown with the arrows in the images. The arrowheads on the top of the cross-sectional profiles indicate major protrusions corresponding to protomer positions. (**e**) A possible structure (left) and a schematic (middle) of twisted-half-spiral ring form. A simulated AFM image obtained from the possible structure is also shown (right). Scale bar, 5 nm.

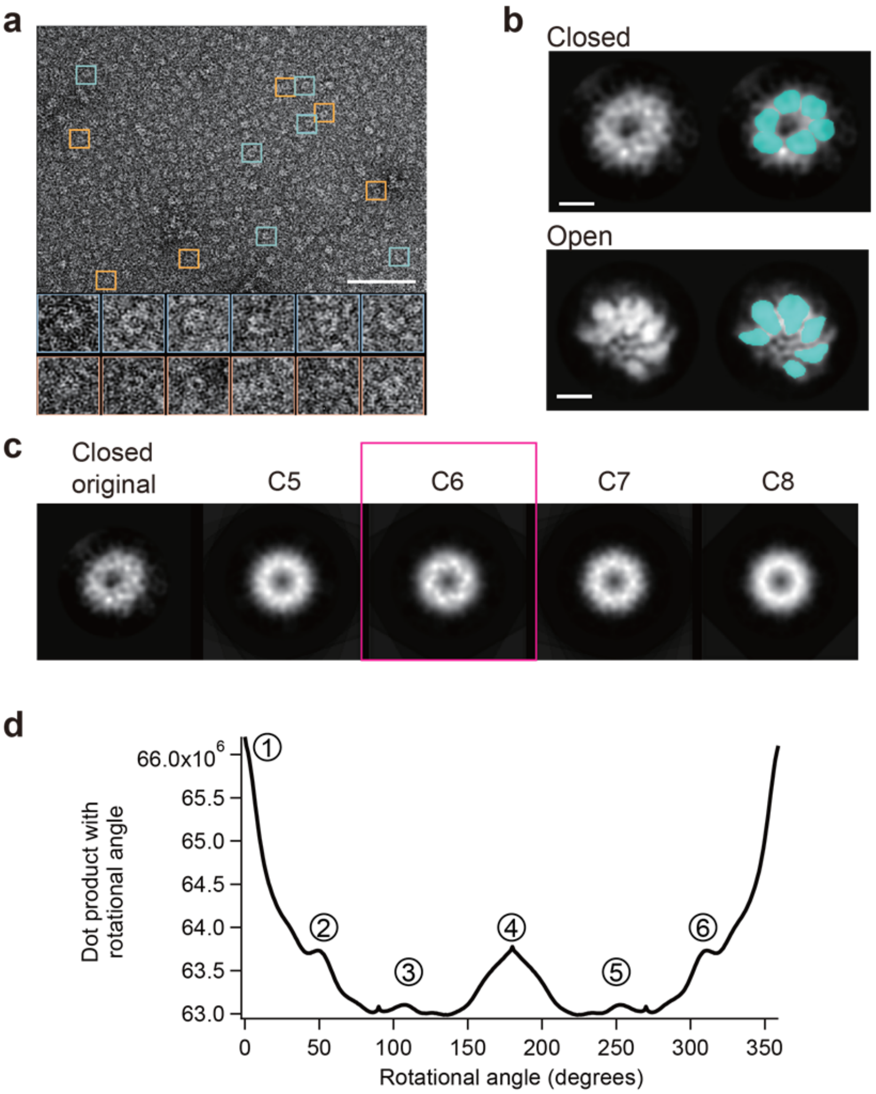

Supplementary Figure 4 | Negative-staining electron microscopy of full-length *T*ClpB particles. (**a**) Representative particles of open and closed forms of full-length *T*ClpB are boxed and thumbnailed with colors of sky blue and orange, respectively. Scale bar, 100 nm. (**b**) 2D class averages of closed and open full-length *T*ClpB particles, in which closed 630 and open 197 particles are averaged. Individual subunits are highlighted in the right panels. Scale bar, 5 nm. (**c**) Rotational symmetries from C5 to C8 are imposed on the closed particle image (original), supposing that the closed particle includes six-hold rotational symmetry. (**d**) Dot product between the original closed particle image and the rotated image as a function of rotational angle. Six peaks were observed, consistent with the hexamer structure.

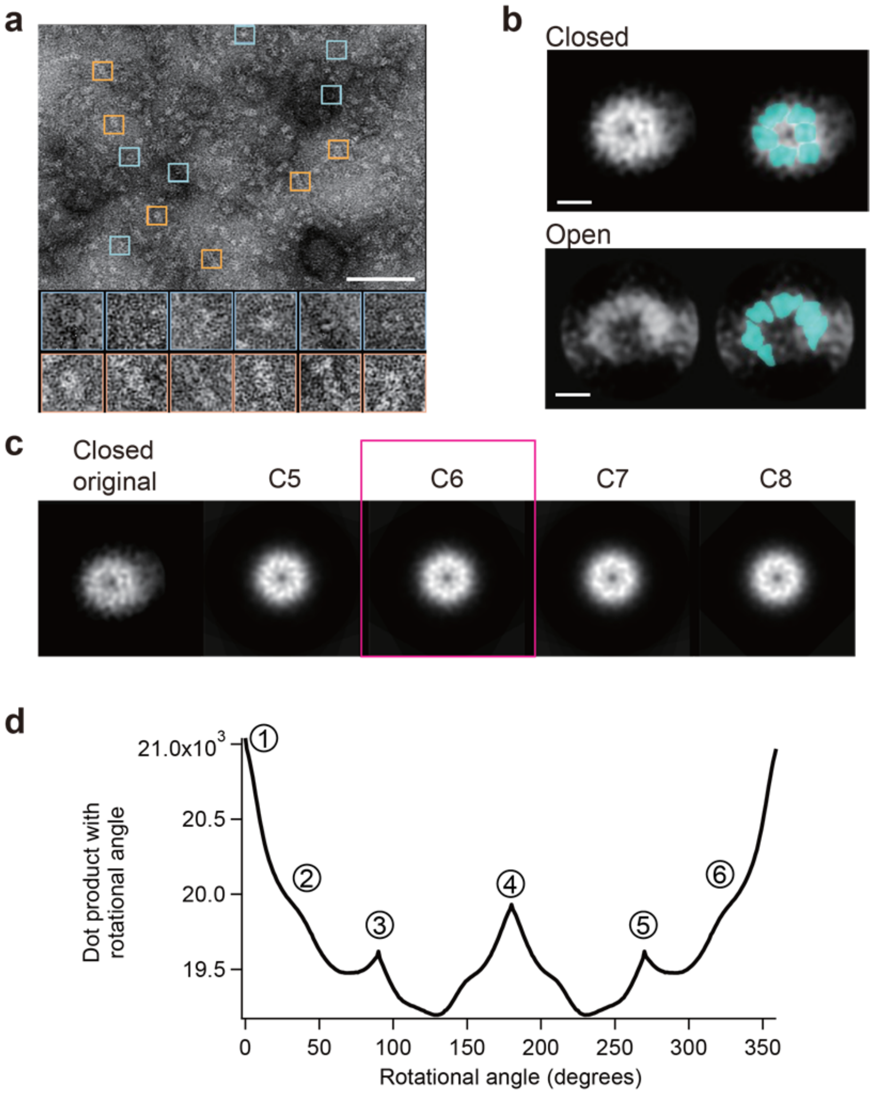

Supplementary Figure 5 | Negative-staining electron microscopy of ΔN-*T*ClpB particles. (**a**) Representative particles of open and closed forms of ΔN-*T*ClpB are boxed and thumbnailed with colors of sky blue and orange, respectively. Scale bar, 100 nm. (**b**) 2D class averages of closed and open ΔN-*T*ClpB particles, in which closed 290 and open 24 particles are averaged. Individual subunits are highlighted in the right panels. Scale bar, 5 nm. (**c**) Rotational symmetries from C5 to C8 are imposed on the closed particle image (original), supposing that the closed particle includes six-hold rotational symmetry. (**d**) Dot product between the original closed particle image and the rotated image as a function of rotational angle. Six peaks were observed, consistent with the hexamer structure.

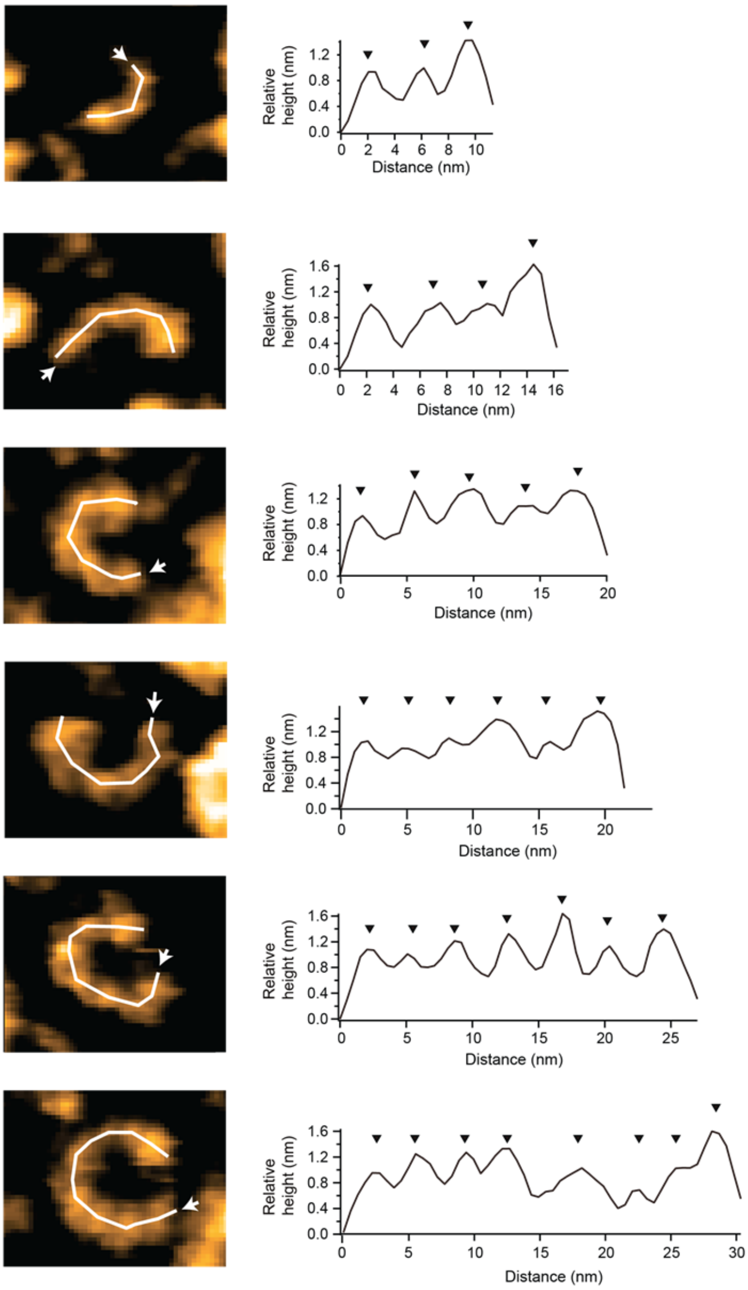

Supplementary Figure 6 | Number of protomers constituting open-form oligomers. (**Left panels**) AFM images of typical open ring forms of ΔN-*T*ClpB. (**Right panels**) The cross sections along the white lines overlaid on the images display the different number of protomers as indicated by the arrowheads above each cross-section.

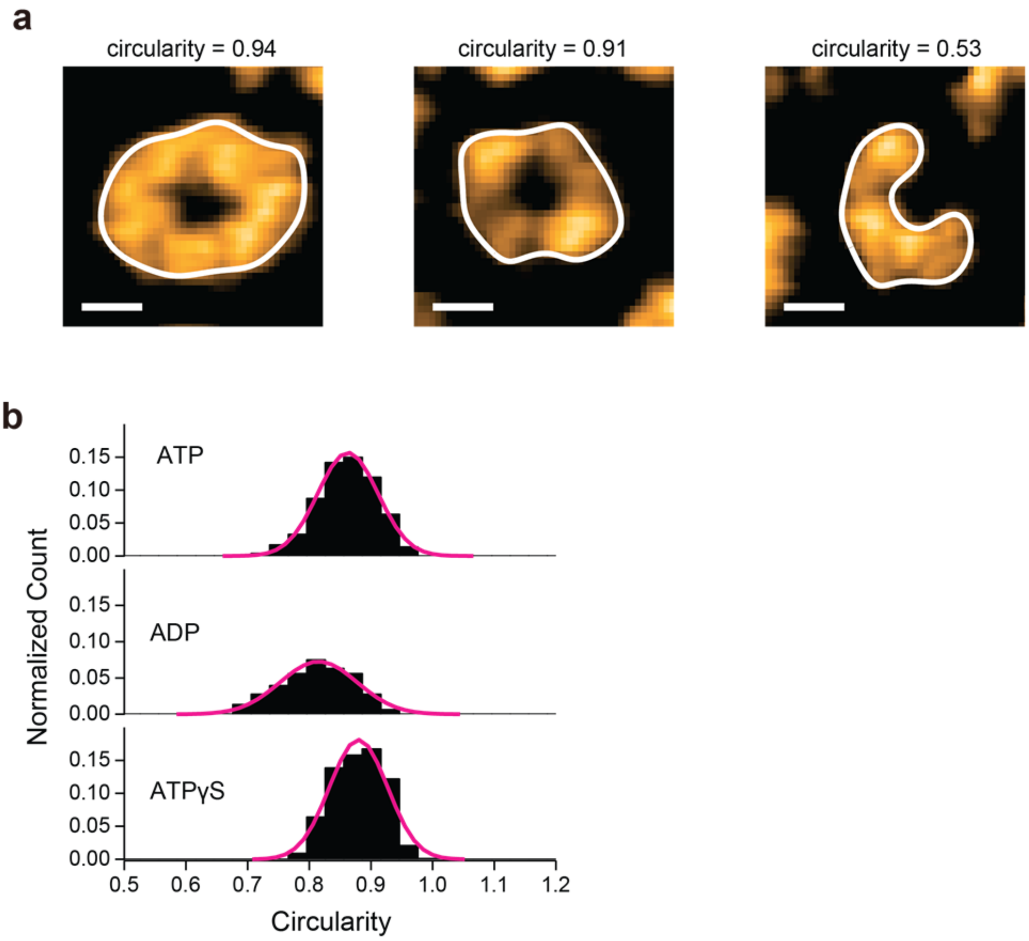

Supplementary Figure 7 | Circularity analysis of ΔN-*T*ClpB oligomers. (**a**) AFM images of round closed (left), distorted closed (middle) and open (right) forms of oligomers encircled with respective outlines (white lines). The circularity is defined by $4\pi S/L^2$, where L and S are the contour length of outline and the area surrounded by the outline, respectively. (**b**) Histograms of circularity measured for closed rings at 1 mM ATP (248 molecules), 1 mM ATPγS (286 molecules) and 1 mM ADP (157 molecules). The center values and widths of fitted Gaussians are 0.86 and 0.075 for ATP, 0.83 and 0.088 for ADP, 0.89 and 0.066 for ATPγS, respectively.

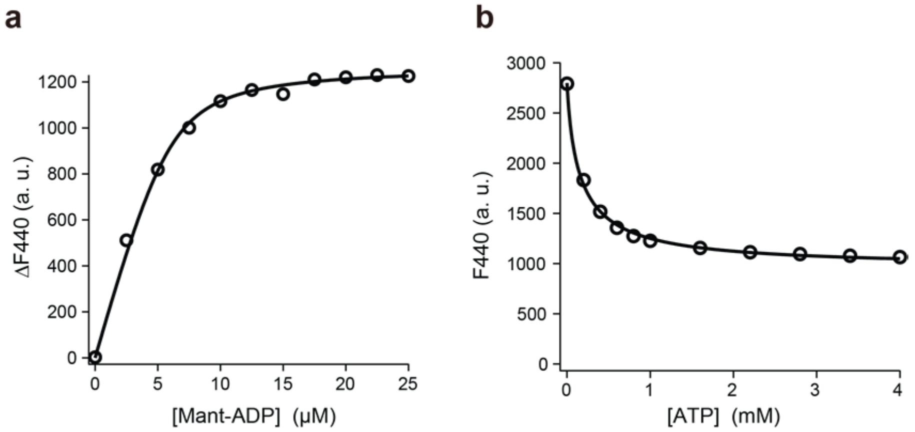

Supplementary Figure 8 | Analysis of ATP binding to ΔN-*T*ClpB. (**a**) Increase of fluorescence intensity of Mant-ADP induced by its mixing with ΔN-*T*ClpB (1 μM as hexamer) at the indicated concentrations of Mant-ADP. The dissociation constant of Mant-ADP was estimated to be 0.64 μM (**b**) ATP binding to ΔN-*T*ClpB estimated by replacement titration. The bound Mant-ADP was replaced with ATP and the resulting fluorescence decrease of Mant-ADP was measured. The dissociation constant of ATP was estimated to be 9.2 μM. All measurements were carried out at 25 °C.

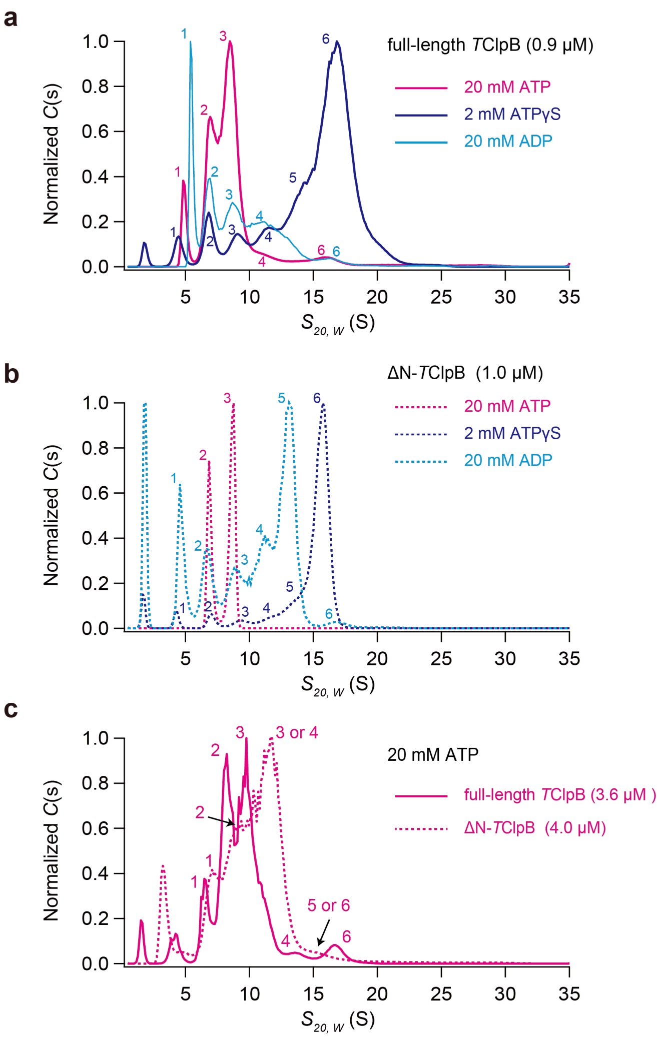

Supplementary Figure 9 | Analysis of *T*ClpB oligomer forms by sedimentation velocity analytical ultracentrifugation (SV-AUC). (**a**) Sedimentation coefficient distributions of full-length *T*ClpB in the presence of 20 mM ATP (magenta line), 2 mM ATPγS (blue line) and 20 mM ADP (turquoise line). The concentration of full-length *T*ClpB was 0.9 µM. (**b**) Sedimentation coefficient distributions of ΔN-*T*ClpB in the presence of 20 mM ATP (magenta line), 2 mM ATPγS (blue line) and 20 mM ADP (turquoise line). The concentration of ΔN-*T*ClpB was 1.0 µM. (**c**) Sedimentation coefficient distributions of full-length *T*ClpB (solid line) and ΔN-*T*ClpB (dotted line) with higher protein concentrations in the presence of 20 mM ATP. The concentrations of full-length *T*ClpB and ΔN-*T*ClpB were 3.6 µM and 4.0 µM, respectively. The assigned number of protomers is indicated near each distribution peak. In 20 mM ATP, the values of sedimentation coefficient shifted to higher values at higher protein concentration for both the full-length and the ΔN-*T*ClpB (**a** and **c**), suggesting large dissociation rates of the oligomers.

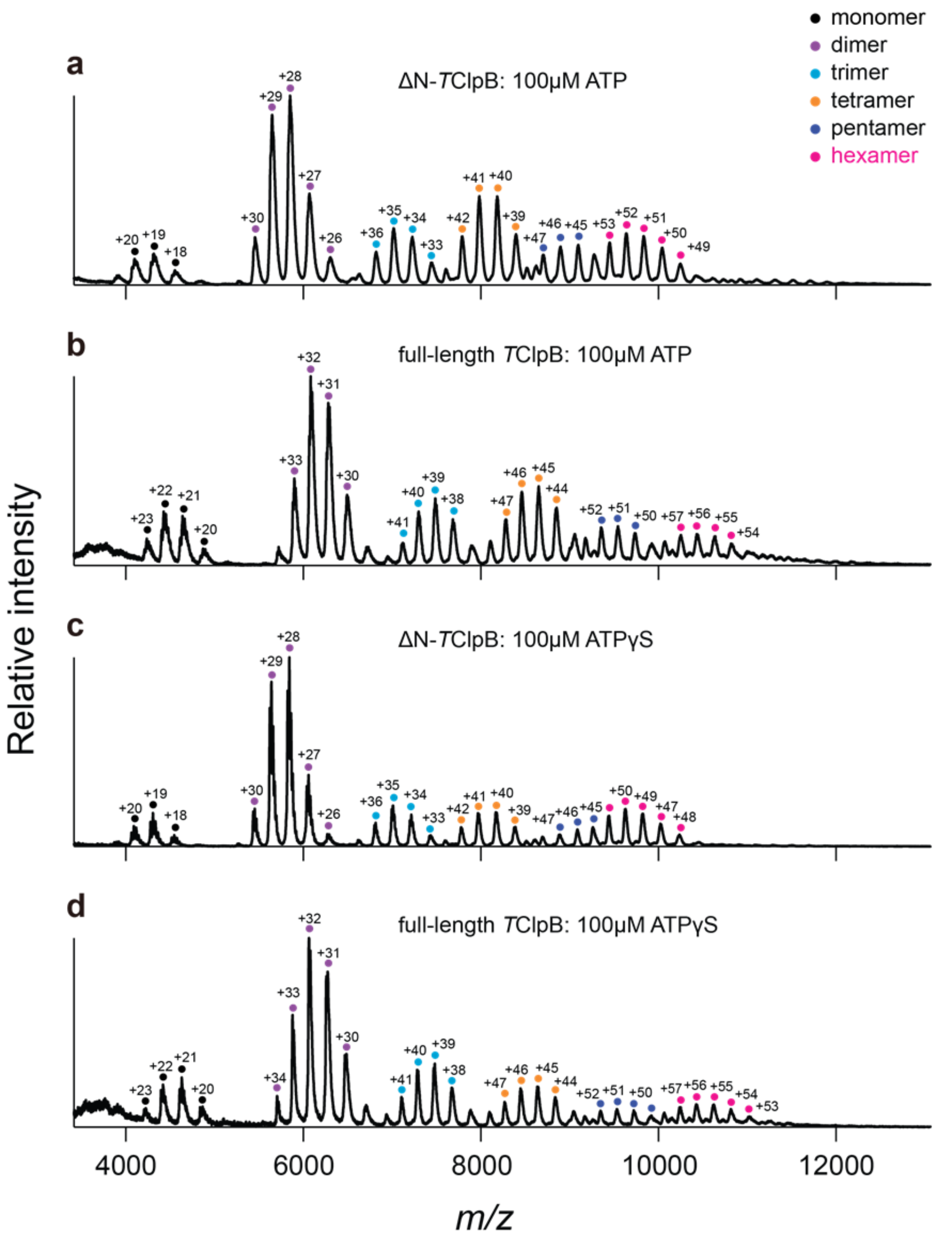

Supplementary Figure 10 | Mass spectra of *T*ClpB. (**a, c**) Mass spectra of ΔN-*T*ClpB in the presence of 100 μM ATP (**a**) and 100 μM ATPγS (**c**). (**b**, **d**) Mass spectra of full-length *T*ClpB in the presence of 100 μM ATP (**b**) and 100 μM ATPγS (**d**). Black, purple, turquoise, orange, blue, and magenta circles correspond to the ion series of monomer, dimer, trimer, tetramer, pentamer and hexamer, respectively. The estimated masses of the oligomers are summarized in **Supplementary Table 1**.

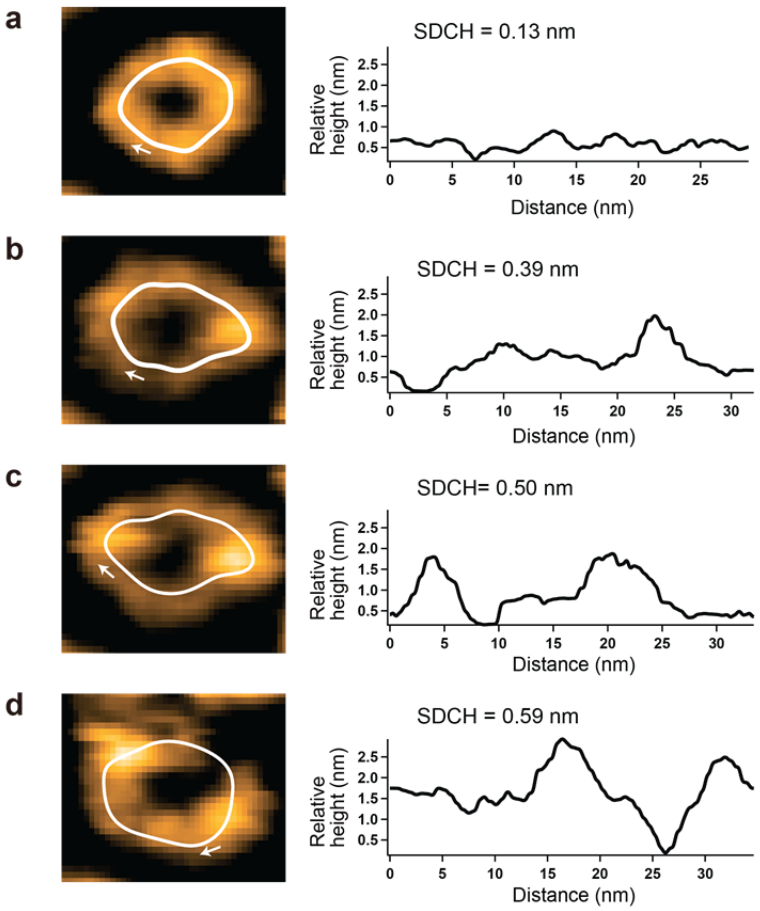

Supplementary Figure 11 | SDCH for different oligomer forms. (**left**) Typical AFM images of round closed (**a**), spiral (**b**), twisted-half-spiral (**c**) and open (**d**) forms. The white lines overlaid on the images show 2D positions where the cross-sectional profiles are measured. (**right**) Cross-sectional profiles along the respective white outline and their SDCH values.

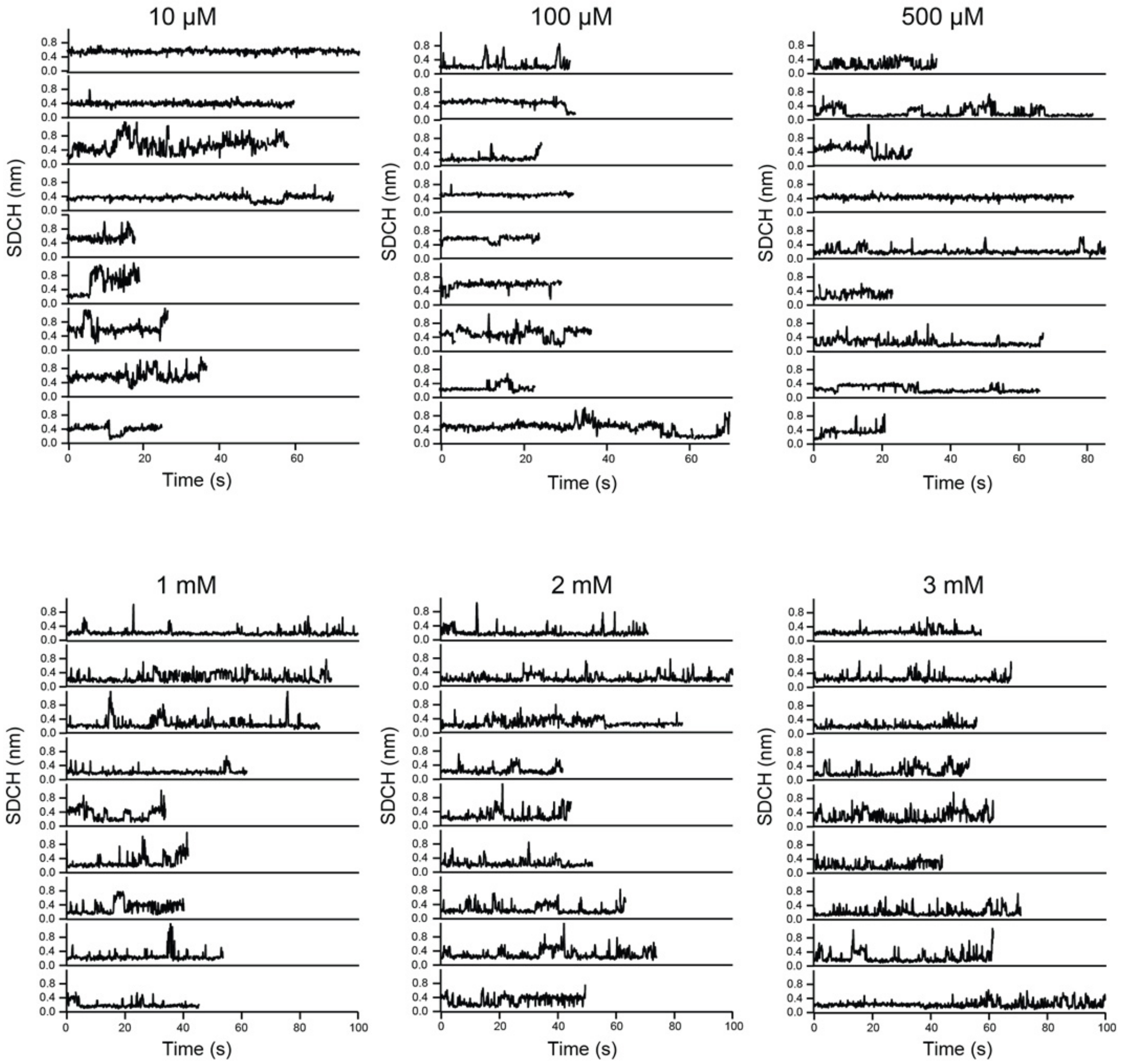

Supplementary Figure 12 | Time courses of changes in SDCH of ΔN-*T*ClpB at different ATP concentrations. Time courses for 9 molecules are shown for each ATP concentration condition.

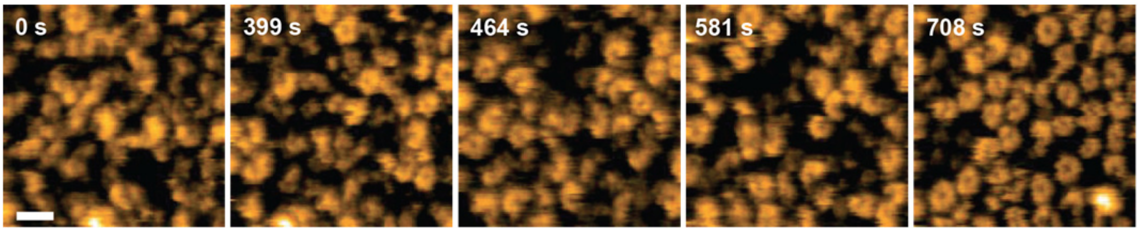

Supplementary Figure 13 | Clipped HS-AFM images showing formation of oligomer rings of ΔN-*T*ClpB after the addition of ATP. During HS-AFM imaging, ATP (3 mM in final) was added at time zero to the sample that had not been preincubated in the presence of ATP. Scale bar, 20 nm. Frame rate, 2 fps.

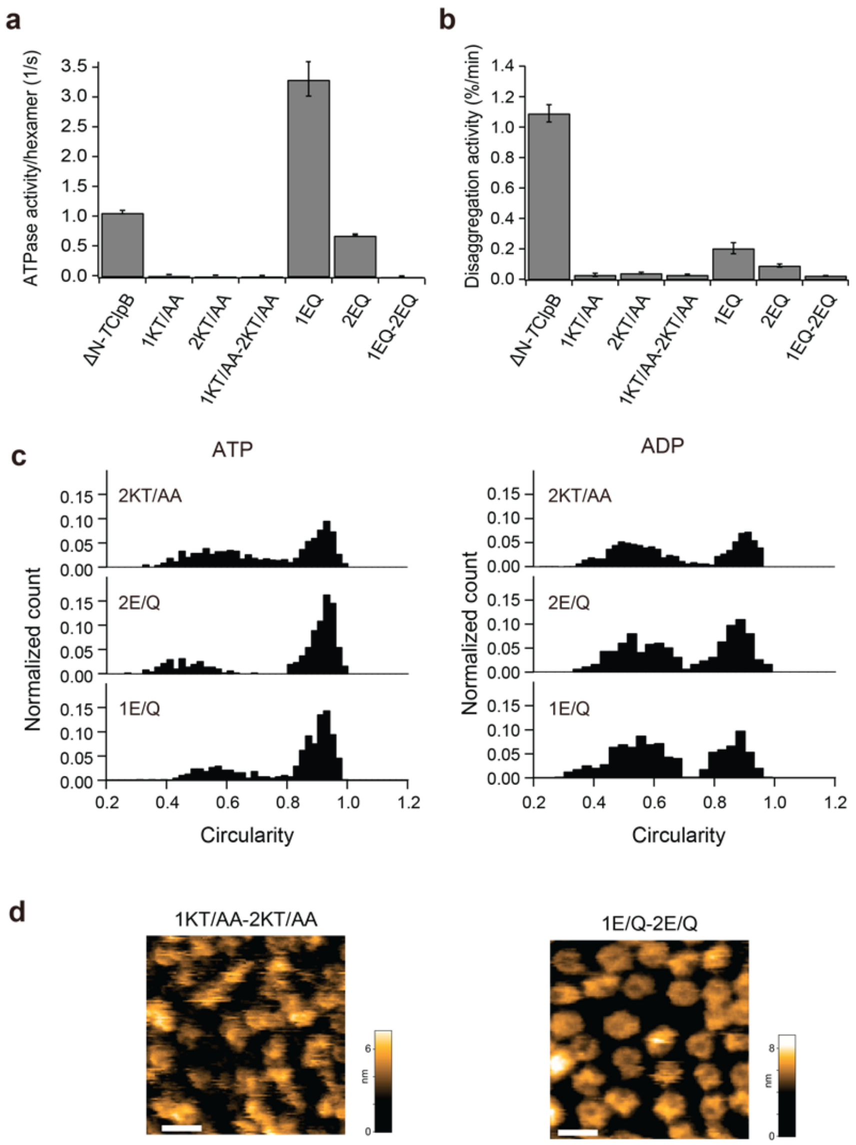

Supplementary Figure 14 | ATPase and disaggregation activities and circularity analysis of the Walker motif mutants. (**a**) ATPase and (**b**) disaggregation activities of ΔN-*T*ClpB and Walker-motifs mutants. Data are mean ± s.d. (**c**) Histograms of circularity measured for Walker-motifs mutants at 1 mM ATP (368, 515, and 1102 molecules for 1E/Q, 2E/Q, and 2KT/AA, respectively) and 1 mM ADP (388, 406, and 394 molecules for 1E/Q, 2E/Q, and 2KT/AA, respectively). (**d**) Typical HS-AFM images of double Walker A (1KT/AA−2KT/AA) and Walker B (1E/Q−2E/Q) mutants observed in the presence of 1 mM ATP. Scale bars, 20 nm. Z color bars, 0 to 7.4 nm for 1KT/AA−2KT/AA and 0 to 9.6 nm for 1E/Q−2E/Q.

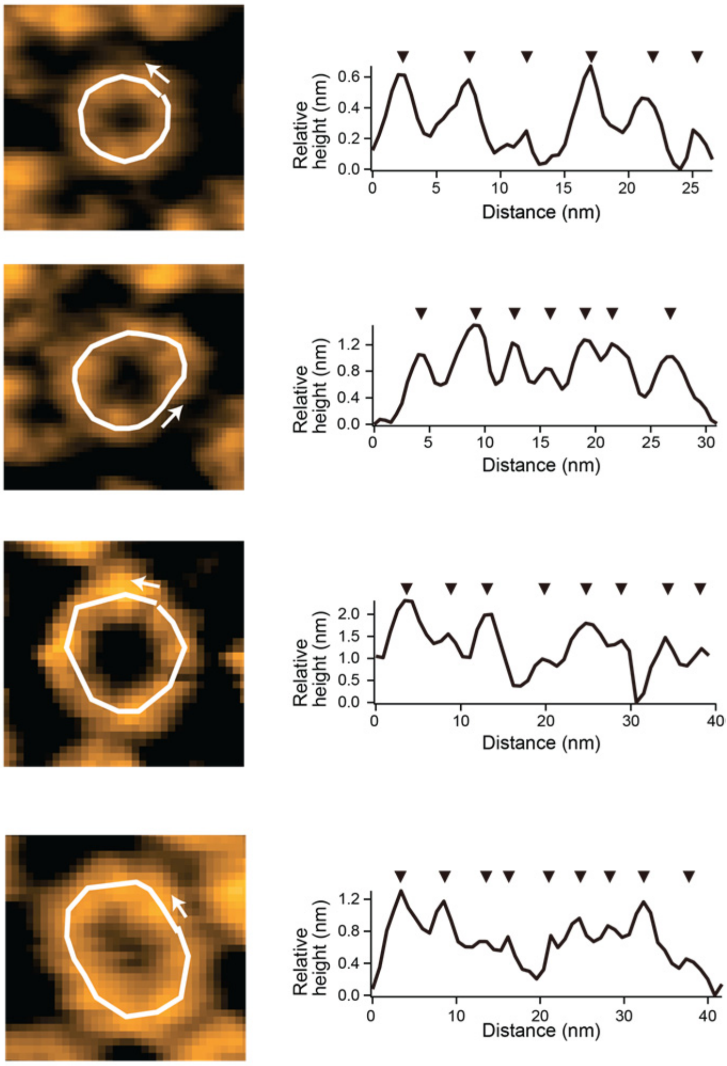

Supplementary Figure 15 | Diverse numbers of protomers contained in closed rings of 2KT/AA mutant. Typical AFM images of closed rings of 2KT/AA mutants with different sizes are shown. The white lines overlaid on the images show 2D positions where the cross-sectional profiles shown in the right panels are measured. The arrowheads pointing the height peaks correspond to the protomer positions.

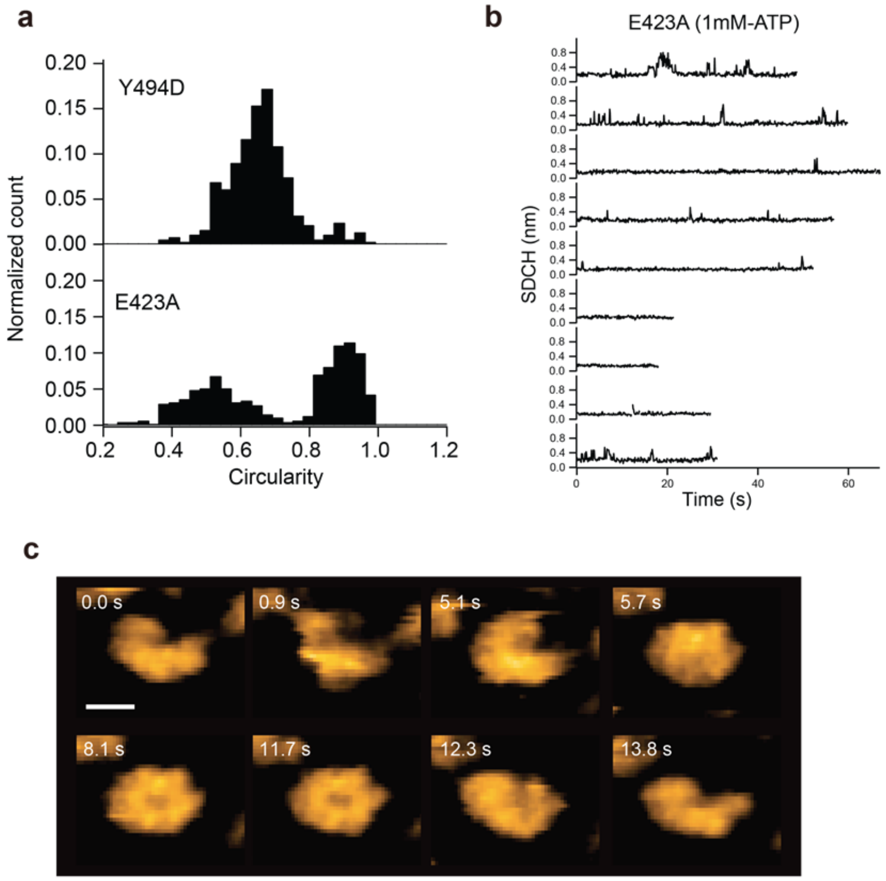

Supplementary Figure 16 | State analysis of activity mutants. (**a**) Histograms of circularity measured for Y494D hyperactive (top, 378 molecules) and E423A repressed (bottom, 472 molecules) mutants. (**b**) Time courses of changes in SDCH of E423A at 1mM ATP. (**c**) Clipped HS-AFM images of Y494D hyperactive mutant, showing transitions between open and closed forms at 1 mM ATP. Scale bar, 10 nm. Frame rate, 3.3 fps.

Supplementary Table 1. Masses of *T*ClpB oligomers determined by NMS

*T*ClpB	Measured (Da)		Theoretical (Da)
**ΔN (ATP)**			
Monomer	81,906	± 47*	81,877
Dimer	163,702	± 60	163,753
Trimer	245,495	± 39	245,630
Tetramer	327,071	± 187	327,506
Pentamer	408,964	± 75	409,383
Hexamer	491,264	± 99	491,259
**Full length (ATP)**			
Monomer	97,343	± 61	97,325
Dimer	194,620	± 52	194,650
Trimer	291,777	± 185	291,975
Tetramer	389,095	± 287	389,300
Pentamer	486,505	± 174	486,625
Hexamer	584,244	± 294	583,950
**ΔN (ATPγS)**			
Monomer	81,799	± 18	81,909
Dimer	163,555	± 5	163,818
Trimer	245,328	± 34	245,727
Tetramer	326,797	± 233	327,636
Pentamer	408,585	± 280	409,545
Hexamer	491,164	± 496	491,454
**Full length (ATPγS)**			
Monomer	97,234	± 15	97,357
Dimer	194,393	± 343	194,715
Trimer	291,807	± 84	292,072
Tetramer	388,907	± 177	389,429
Pentamer	486,281	± 274	486,787
Hexamer	583,923	± 341	584,144

*Values shown are the means ± S.D. of triplicated assays.

Supplementary Table 2. Primers used for generation of *T*ClpB mutants

Name	Sequence
TClpB_ΔN-f	5'-AGGAGATATACATATGCAGACGGAACACGC-3'
TClpB_ΔN_Q142C-f	5'-GGAATTCCATATGTGTACAGAACACGCGG-3'
TClpB_1KT/AA-f	5'-GAGCCCGGCGTGGGGGCCGCGGCCATCGTGGAGGGCCTG-3'
TClpB_1KT/AA-r	5'-CAGGCCCTCCACGATGGCCGCGGCCCCCACGCCGGGCTC-3'
TClpB_1E/Q-f	5'-CATCCTCTTCATTGATCAGCTCCACACCGTGGTGGGGGCAG-3'
TClpB_1E/Q-r	5'-CTGCCCCCACCACGGTGTGGAGCTGATCAATGAAGAGGATG-3'
TClpB_2KT/AA-f	5'-CCTCGGGCCCACGGGGGTGGGGGCCGCGGAGCTCGCCAAGAC-3'
TClpB_2KT/AA-r	5'-GTCTTGGCGAGCTCCGCGGCCCCCACCCCCGTGGGCCCGAGG-3'
TClpB_2E/Q-f	5'-CATCCTCTTTGATCAGATTGAGAAGGCCCACCCCGACG-3'
TClpB_2E/Q-r	5'-CGTCGGGGTGGGCCTTCTCAATCTGATCAAAGAGGATG-3'
TClpB_E423A-f	5'-TTTAAAGAAGGCGAAGGACCCGGAC-3'
TClpB_E423A-r	5'-GCCTTCTTTAAAGCCTCCCGCTCAATC-3'
TClpB_Y494D-f	5'-GATGGGGAGCTTCCCAAGCTTGAGGCCGAGGTG-3'
TClpB_Y494D-r	5'-AAGCTCCCCATCGCGGAGCTCGGCGGCCCGGTTC-3'
T7r	5'-CTAGTTATTGCTCAGCGGTGGCAG-3'
