# Supplementary material for: Dynamic structural states of ClpB involved in its disaggregation function
Source: Nat Commun. 2018 Jun 1;9:2147. doi: 10.1038/s41467-018-04587-w (PMC5984625; doi:10.1038/s41467-018-04587-w)
Supplement: Supplementary file 3 — Description of Additional Supplementary Information [file 41467_2018_4587_MOESM3_ESM.docx]

**Description of Additional Supplementary Files**

File Name: Supplementary Movie 1

Description: Dynamics of ΔN-TClpB oligomer at 10 μM ATP.

File Name: Supplementary Movie 2

Description: Dynamics of ΔN-TClpB oligomer at 100 μM ATP.

File Name: Supplementary Movie 3

Description: Dynamics of ΔN-TClpB oligomer at 500 μM ATP.

File Name: Supplementary Movie 4

Description: Dynamics of ΔN-TClpB oligomer at 2 mM ATP.

File Name: Supplementary Movie 5

Description: Dynamics of ΔN-TClpB oligomer at 3 mM ATP.

File Name: Supplementary Movie 6

Description: Oligomerization of ΔN-TClpB after the addition of 3 mM ATP to the protein not preincubated beforehand in the presence of ATP.

File Name: Supplementary Movie 7

Description: Subunit shuffling of ΔN-TClpB at 2 mM ATP.

File Name: Supplementary Movie 8

Description: Subunit shuffling of ΔN-TClpB at 2 mM ATP.

File Name: Supplementary Movie 9

Description: Open-form oligomer of Y494D hyperactive mutant at 1 mM ATP.
